# The human microplastic burden and brain health: From measurement to pathophysiology and removal

**DOI:** 10.61373/bh026p.0006

**Published:** 2026-05-05

**Authors:** Julio Licinio, Charlotte Steenblock, Nicholas Fabiano, Stefan R. Bornstein, Ma-Li Wong

**Affiliations:** 1 Genomic Press, New York, New York 10036, USA;; 2 Department of Internal Medicine III, University Hospital Carl Gustav Carus, Technische Universität Dresden, 01307 Dresden, Germany;; 3 University of Ottawa, Department of Psychiatry, Ottawa, Ontario K1H 8L6, Canada;; 4 School of Cardiovascular and Metabolic Medicine and Sciences, Faculty of Life Sciences & Medicine, King’s College London, London WC2R 2LS, UK;; 5 Department of Endocrinology, Diabetology and Clinical Nutrition, University Hospital Zurich (USZ) and University of Zurich (UZH), 8091 Zurich, Switzerland

**Keywords:** Microplastics, nanoplastics, brain health, ultra-processed foods, neuroinflammation

## Abstract

Microplastics and nanoplastics now contaminate every human compartment examined, including blood, placenta, atheromatous plaque, and brain tissue. The brain warrants particular attention. Decedent brain tissue has been shown to carry microplastic concentrations seven to thirty times higher than liver or kidney, with a 50% rise documented between 2016 and 2024, and the highest burdens observed in donors with diagnosed dementia. Animal data confirm that nanoscale particles cross the blood-brain barrier within hours of oral exposure. Ultra-processed foods, which now account for more than half of caloric intake in the United States, serve as high-throughput delivery vehicles for microplastics via packaging migration, mechanical processing, and contamination. They have themselves been independently linked in large prospective cohorts to depression, anxiety, cognitive decline, stroke, and dementia. The convergence of high tissue burden, biologically plausible mechanism, and a population-scale modifiable exposure vehicle defines a Brain Health emergency. We outline what is known, what is not yet measurable, and where the field must move next, with attention to vulnerable populations including pregnant women, children, and patients with established neurological or cardiovascular disease.

In a new journal aimed at promoting brain health, why are we focus-ing on micro- and nanoplastics to start with? Because brain health can-not be achieved in brains that are increasingly soaked in microplastics, or, more precisely, nanoplastics. We use “microplastics” throughout this Perspective as the field’s prevailing term, although “nanoplastics” is the more accurate descriptor for the particles of greatest biological concern. Those that cross the blood-brain barrier, accumulate in brain tissue, and lodge within the intracellular compartment of placenta and atheroma are predominantly nanoscale. The reality is more nano than micro.

## The human microplastic burden

We use the term *microplastic burden* to describe the cumulative load of microplastic and nanoplastic particles deposited within the human body. The term *load* is already in use in environmental science, applied to ecosystems, water systems, and waste streams. *Burden* is the closer fit for clinical contexts, capturing what we mean when we discuss tumor burden, viral burden, or atherosclerotic burden: a measurable accumulation that varies between individuals, tracks with exposure, and plausibly predicts outcomes.

The human microplastic burden is not a single number. It is tissue-specific, polymer-specific, size-specific, and dynamic. It rises with cumulative exposure and may, in principle, be reduced by intervention. Defining it operationally, measuring it reliably, and learning what it predicts clinically are the three challenges that organize the rest of this Perspective.

## Why the brain

A plastic spoon’s worth of polymer, lodged in the human brain. The image, sharp enough to anchor an editorial and the cover article on human microplastic removal in our sister journal, *Brain Medicine*, last year (1, 2), and arresting enough to generate worldwide attention, is more than rhetoric. It is a measurement. Nihart and colleagues, in work published in *Nature Medicine*, found that decedent human brain tissue carries microplastic concentrations seven to thirty times higher than matched liver or kidney samples, and that the burden rose by approximately 50% be-tween 2016 and 2024 (3). The cohort with documented dementia diagnoses carried the heaviest load. Polyethylene predominated, presenting largely as nanoscale, shard-like fragments.

Does this finding stand alone? Hardly. Microplastics have been quantified in human blood (4), localized within the intracellular compartment of the placenta (5), and detected within atheromatous plaque, where their presence was associated with a roughly fourfold increase in the composite risk of myocardial infarction, stroke, or death over thirty-four weeks of follow-up (6). The Marfella signal is a cerebrovascular signal as much as it is a cardiac one. Stroke is, after all, a brain outcome.

How do these particles reach the brain? In animal models, the answer is becoming clear. Kopatz and colleagues showed that polystyrene nanoparticles administered orally to mice cross the blood-brain barrier within two hours, and that the biomolecular corona acquired in transit is what enables passage (7). Larger particles do not cross. Nanoscale particles do.

We are looking at an organ where the highest measured concentrations of microplastics meet the most consequential clinical endpoints in medicine: cognition, mood, stroke, and dementia. Treating this as a peripheral environmental concern, when the relevant peripheral organs carry less of the contaminant than the central one, has become difficult to defend.

## What we cannot yet precisely measure

Readers of the Nihart paper can easily access the published mattersarising correspondence in the same journal, which raised legitimate questions about contamination control and analytical validation. That exchange is healthy science. It also illustrates the central problem of the field: we do not yet have validated, reproducible, polymer-specific measurement methods that the broader scientific community accepts as a gold standard.

Pyrolysis gas chromatography-mass spectrometry, attenuated total reflectance Fourier-transform infrared spectroscopy, Raman microspectroscopy, and electron microscopy each have specific strengths and known limitations. Particle counts versus mass quantification yield different answers. Detection thresholds vary across laboratories. Blank controls and procedural contamination remain difficult to fully characterize, particularly for ubiquitous polymers such as polyethylene, which are present throughout any analytical environment.

Brain tissue compounds these difficulties. The matrix is lipid-rich, which interferes with several digestion protocols. Regional heterogeneity is poorly mapped. Does a frontal cortex measurement generalize to deeper structures? We do not know. Post-mortem blood content in tissue samples is non-negligible, so investigators must distinguish true parenchymal penetration from vascular contamination. The brain is one of the most analytically demanding organs for quantifying microplastic burden, and it is the organ where the answer matters most.

We cannot reduce a burden we cannot quantify. We cannot rank polymers by harm without a shared measurement standard. We cannot test interventions without a biomarker that responds to them. The measurement problem is the precondition for everything else the field hopes to do, including the removal strategies discussed below.

## Pathophysiology in search of a hierarchy

**Figure 1.**
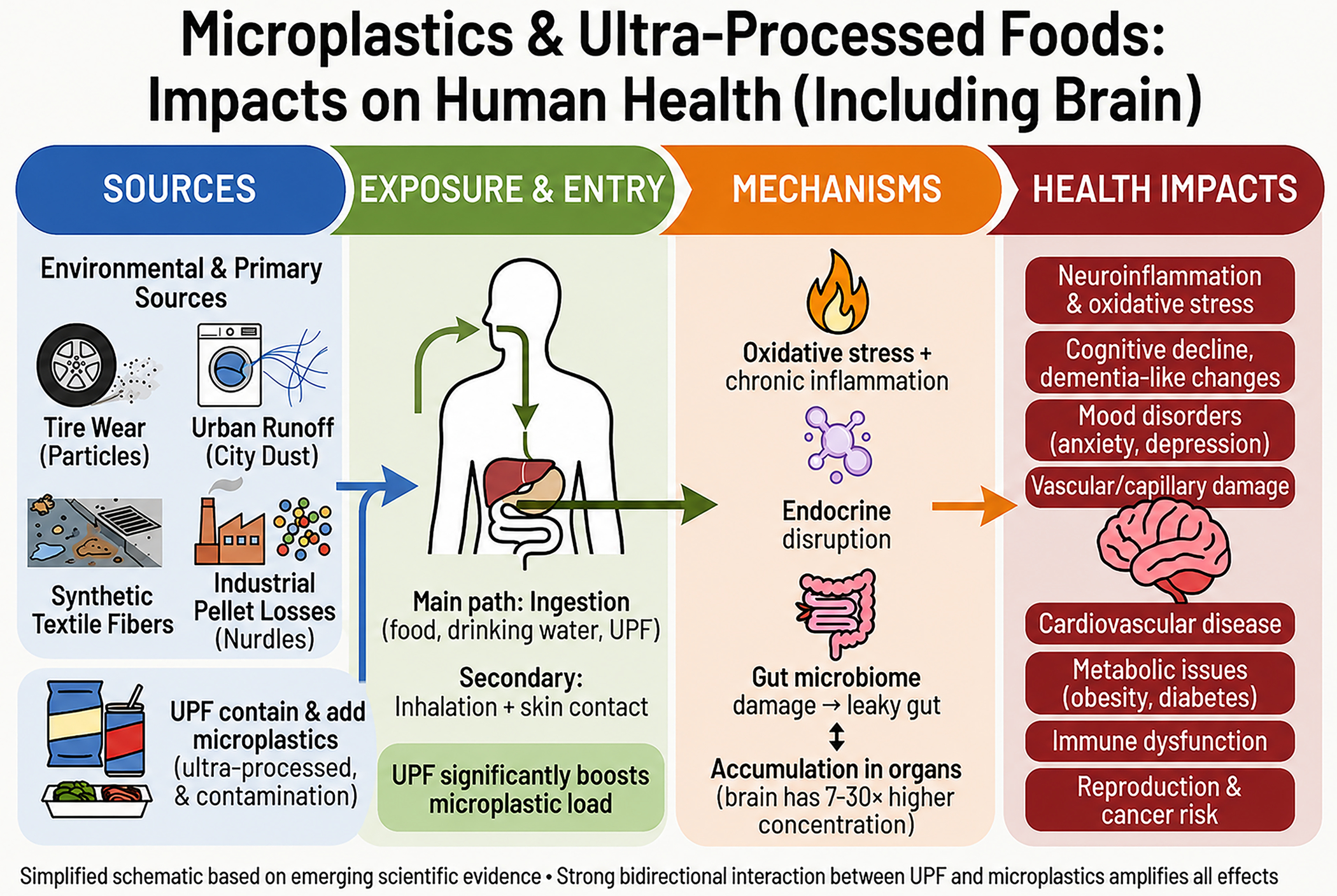
The human microplastic burden and brain health. Schematic and simplified overview of selected sources, exposure routes, mechanisms, and health impacts associated with microplastic and nanoplastic accumulation in the human body. Ultra-processed foods (UPF) both contain and add microplastics, and significantly boost overall intake. Ingestion is the principal exposure route. Proposed mechanisms include oxidative stress and chronic inflammation, endocrine disruption, gut-microbiome injury, and tissue accumulation, with brain concentrations approximately seven to thirty times higher than liver or kidney. Associated health impacts span neuroinflammation, cognitive decline, mood disorders, cardiovascular and metabolic disease, and possible reproductive harm and cancer risk. The figure highlights some, not all, aspects of this rapidly evolving field. Figure generated by JL through multiple iterations with Gemini and Grok, then edited in Adobe Photoshop. © Julio Licinio 2026.

The accompanying schematic shows four mechanistic pathways through which microplastics plausibly damage the brain: oxidative stress and chronic inflammation, endocrine disruption, gut-microbiome injury with consequent gut-brain signaling, and vascular and capillary damage (Figure 1). Each pathway is supported by animal and in vitro evidence, and each converges on outcomes that brain-health professionals recognize from clinical practice. Neuroinflammation, vascular cognitive impairment, mood dysregulation through inflammatory and microbiome-mediated routes, and endocrine perturbation of neurodevelopment are familiar territory. We see them, in different forms, in our patients every week.

The harder question is which polymers, at which sizes, in which tis-sues, drive which mechanisms most strongly. Polyethylene predominates in brain. Polyvinyl chloride is also prominent in atheromatous plaque (6). Polystyrene crosses the blood-brain barrier most readily in animal models (7). These are not interchangeable findings. A general “microplastic” category, useful as it has been for raising the alarm, no longer suffices as a guide to intervention. The field needs polymer-specific risk stratification: a ranking, defensible across laboratories, of which polymers in which size fractions at which concentrations in which tissue compartments warrant the most urgent attention.

We have not yet created that ranking. Producing it will require the measurement infrastructure described above, animal mechanistic studies that compare polymers head to head rather than in isolation, and human cohort studies that link polymer-specific tissue burdens to clinical outcomes. None of this work is impossible. None of it will happen quickly. In the interim, the bidirectional relationship between microplastics and ultra-processed foods (UPF), articulated by Fabiano, Luu, Puder, and Marx (8), has become an organizing principle. Microplastics enter the body predominantly through ingestion. UPF are a dominant ingestion vehicle. Each amplifies the other. That framing is what makes the next section possible. UPF are industrial formulations built from extracted food substances and additives, with little or no intact whole food. They are group 4 of the NOVA classification, a system developed by Carlos Monteiro and colleagues that sorts foods by the degree of industrial processing rather than by nutrient content (9). Soft drinks, packaged snacks, instant noodles, mass-produced breads, and ready-to-heat meals are the familiar examples.

## Ultra-processed food as the modifiable input

The case for UPF as an independent risk factor for adverse brain outcomes has accumulated faster than the microplastic literature, and from larger samples. Lane and colleagues, in a meta-analysis of 385,541 participants, found that higher UPF consumption was associated with a 53% increase in the odds of common mental disorder symptoms, a 44% increase for depression specifically, and a 48% increase for anxiety. Prospective data within the same analysis showed a 22% increase in the risk of subsequent depression (10).

The neurological evidence is similarly consistent. Li and colleagues, drawing on the UK Biobank, reported in Neurology that higher consumption of UPF was prospectively associated with an increased risk of dementia, with substitution of unprocessed or minimally processed foods conferring a lower risk (11). Bhave and colleagues, in the REGARDS cohort (Reasons for Geographic and Racial Differences in Stroke), reported that a 10% increase in relative UPF intake was associated with a 16% increase in the risk of cognitive impairment and an 8% increase in the risk of stroke. These associations held independently of adherence to the Mediterranean, DASH (Dietary Approaches to Stop Hypertension), or MIND (Mediterranean-DASH Intervention for Neurodegenerative Delay) dietary patterns (12). The food-processing variable does work that established dietary frameworks do not capture.

Why? The UPF category is heterogeneous, and several mechanisms have been proposed: nutrient displacement, additive load, glycemic disruption, inflammatory effects of emulsifiers, and refined oils. All of these matter. The microplastic content of UPF, deriving from packaging migration during heating and storage, mechanical wear during industrial processing, and downstream contamination, is one of the few candidate mechanisms that explains why the food-processing variable retains its predictive power after adjustment for traditional nutritional patterns. Polymer-rich packaging touches UPF at every stage of their production and distribution. Whole foods, by definition, encounter much less of it.

This convergence between dietary epidemiology and exposure science also reframes our thinking about the boundary between physical and mental health. As Fabiano has argued, that boundary has always been more administrative than biological (13). Microplastics do not respect it. The same particles that lodge in atheroma also reach the brain, and the same dietary exposures that raise risk for cardiovascular disease also raise risk for depression and dementia. We are not looking at parallel problems requiring parallel solutions. We are looking at one problem with many clinical faces.

This framing has practical consequences. Population-scale microplastic exposure reduction, in the absence of any validated clinical removal modality, is currently achievable only by reducing UPF consumption. That is not a trivial intervention, and it is not without controversy in nutrition science, but it is the one lever the field has at present that operates at the scale of the problem.

## Removal as a plausible frontier

Has the question of whether microplastics can be removed from the human body moved from speculation to active investigation? Within the past year, yes. Bornstein and colleagues reported that therapeutic apheresis can extract material consistent with microplastic particles from human plasma, offering the first credible demonstration that an established clinical modality may engage these particles in vivo (14). The mechanism is biologically plausible. The signal is consistent with removal. The clinical infrastructure already exists in tertiary centers worldwide. On present evidence, this is the most promising candidate intervention the field has produced.

Can we say with certainty that apheresis definitively reduces microplastic burden? Not yet, and the reason returns the discussion to the measurement problem. Without validated, reproducible, polymer-specific quantification methods applicable to plasma and tissue, no intervention can be confirmed to have worked in the strict sense (2). That provisional status is not a weakness of the apheresis approach. It is a feature of a field operating ahead of its own analytical tools. The principled response is to invest in the measurement standards that would let the apheresis signal be confirmed, refined, or revised, rather than to dismiss promising data because the gold-standard methods do not yet exist.

The brain-specific question compounds this. The blood-brain barrier is asymmetric. Nanoscale particles enter the brain within hours of oral exposure, but their efflux pathways are unmapped, and the kinetic relationship between circulating burden and central nervous system burden has not been established. Will peripheral plasma clearance reduce brain bur-den in parallel? We do not yet know. Answering will require brainrelevant biomarkers the field does not yet possess. Surrogate measures, potentially including cerebrospinal fluid sampling in carefully selected patient populations and advanced imaging modalities calibrated against tissue standards, will need to be developed.

Scalability is the third constraint. Apheresis, even granting its full promise, is resource-intensive and cannot be deployed at population scale. The most exposed and most vulnerable populations, including pregnant women, children, patients with established neurological or cardio-vascular disease, and workers with high occupational exposure, will re-quire approaches that can reach them where they live and work. The research priority is therefore parallel rather than sequential: validate measurement, confirm the apheresis signal against that standard, and develop scalable alternatives matched to polymer specificity, tissue compartment, and patient population. We see this as the next chapter of the work Bornstein and colleagues have made possible, not as a critique of it.

## Vulnerable populations

We routinely care for the populations in which microplastic exposure is most consequential. The placental work of Ragusa and colleagues, demonstrating microplastic localization within the intracellular compartment of human placenta, implies fetal exposure during the most vulnerable window of neurodevelopment (5). Children, with developing blood-brain barriers, longer cumulative exposure horizons, and higher per-kilogram intake than adults, face a lifetime burden trajectory that to-day’s adult cohorts cannot predict. Patients with established cerebrovascular disease, in whom the Marfella stroke signal becomes most clinically relevant, are already in our clinics. So are patients with neurodegenerative disease, in whom the Nihart finding of disproportionately high brain burden in dementia donors raises a question that will not go away: are these particles passenger, accelerator, or contributor?

Workers with high occupational inhalation exposure, including those in plastics manufacturing, recycling, textile production, and construction, represent a quantifiable natural experiment that the field has not yet exploited. These cohorts could provide dose-response estimates that ambient population studies cannot.

None of these groups can be acted upon clinically today. That is not the point. The point is that the population-scale microplastic question has already arrived in the brain-health clinic, in the form of patients whose exposures are higher, whose vulnerability windows are wider, or whose underlying disease is most plausibly modifiable by exposure reduction. Identifying who these patients are, characterizing their burden when methods permit, and beginning to think about stratified interventions are the proper concerns of a conceptually novel journal, *Brain Health*.

## Concluding remarks

The diagram accompanying this Perspective is intentionally simplified (Figure 1). The science is not. Three concrete priorities follow from the evidence reviewed above. First, we need validated, reproducible, polymer-specific measurements of burden in brain tissue and surrogate compartments, without which neither risk stratification nor intervention validation is possible. Second, mechanism studies must move from generic microplastic toxicology to polymer-specific, size-specific, tissue-specific ranking of harm. Third, removal strategies must be developed and tested against brain-relevant biomarkers, with apheresis serving as the proof-of-principle anchor for a wider portfolio of approaches matched to scale.

The plastic spoon image (1) is arresting because it also reflects the conditions under which our patients now live. *Brain Health*, as a journal and as a field, is positioned to convene the conversation between toxicology, nutrition, neurology, and psychiatry that this evidence base now demands. None of these disciplines can address the microplastic question alone. But we can certainly work together to promote brain health by decreasing microplastic load.
